# Roles of METTL3 in cancer: mechanisms and therapeutic targeting

**DOI:** 10.1186/s13045-020-00951-w

**Published:** 2020-08-27

**Authors:** Chengwu Zeng, Wanxu Huang, Yangqiu Li, Hengyou Weng

**Affiliations:** 1 Bioland Laboratory (Guangzhou Regenerative Medicine and Health Guangdong Laboratory), Guangzhou, 510005 China; 2 Institute of Hematology, School of Medicine, Key Laboratory for Regenerative Medicine of Ministry of Education, Jinan University, Guangzhou, 510632 China; 3 The Fifth Affiliated Hospital of Guangzhou Medical University, Guangzhou, 510700 China; 4 Guangzhou Institutes of Biomedicine and Health, Chinese Academy of Sciences, Guangzhou, 510530 China

**Keywords:** RNA modification, METTL3, m^6^A, Cancer, Non-coding RNA, Drug discovery

## Abstract

N^6^-methyladenosine (m^6^A) is the most abundant mRNA modification and is catalyzed by the methyltransferase complex, in which methyltransferase-like 3 (METTL3) is the sole catalytic subunit. Accumulating evidence in recent years reveals that METTL3 plays key roles in a variety of cancer types, either dependent or independent on its m^6^A RNA methyltransferase activity. While the roles of m^6^A modifications in cancer have been extensively reviewed elsewhere, the critical functions of METTL3 in various types of cancer, as well as the potential targeting of METTL3 as cancer treatment, have not yet been highlighted. Here we summarize our current understanding both on the oncogenic and tumor-suppressive functions of METTL3, as well as the underlying molecular mechanisms. The well-documented protein structure of the METTL3/METTL14 heterodimer provides the basis for potential therapeutic targeting, which is also discussed in this review.

## Introduction

**Fig. 1:**
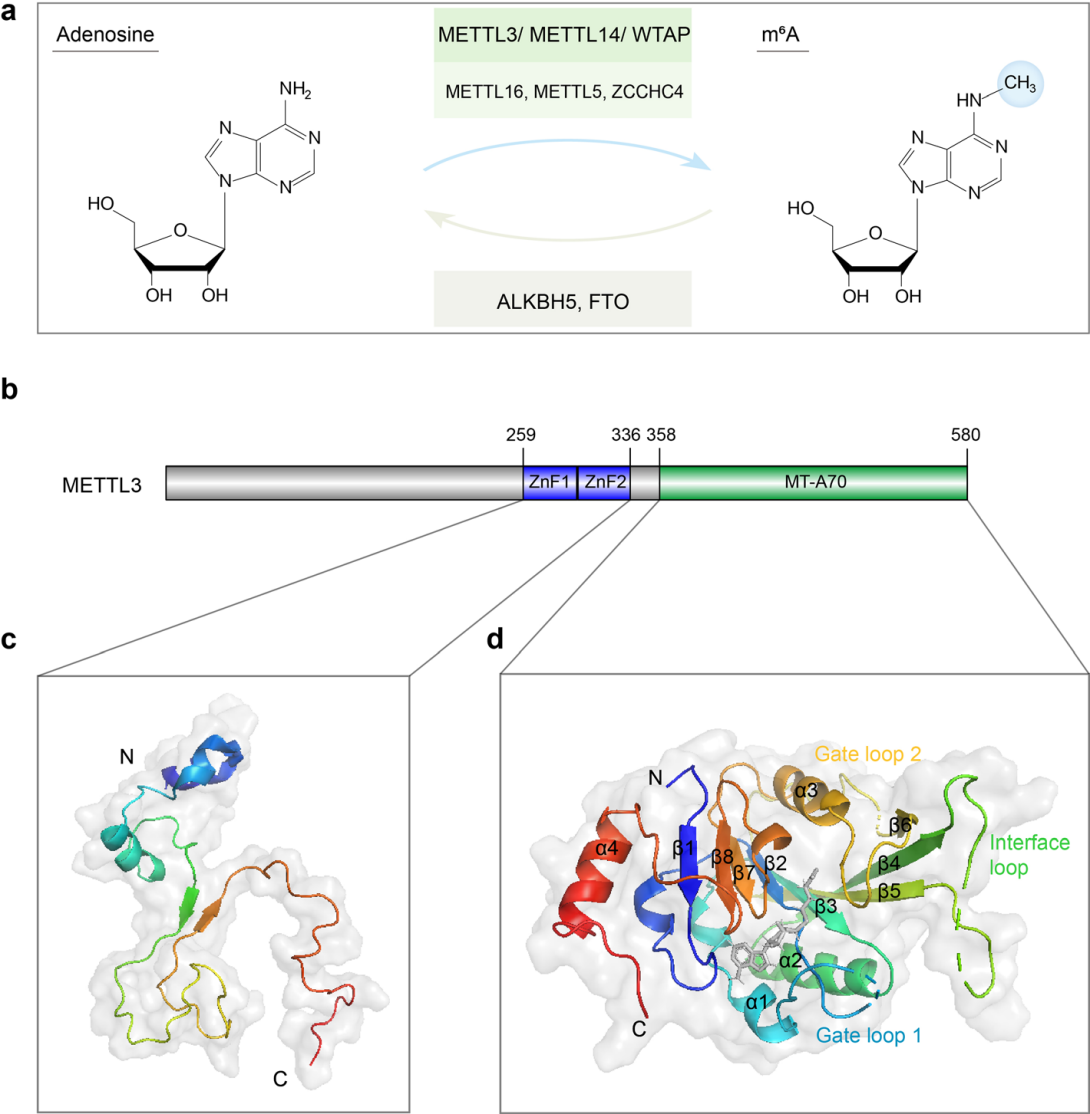
m^6^A RNA methylation and the structure of METTL3, the catalytic subunit of the m^6^A methyltransferase complex. **a** The writers and erasers of the dynamic m^6^A modification. **b** Schematic domain structure of METTL3. **c** Structure of the zinc finger domain (ZFD) of METTL3 (PDB ID: 5yz9). **d** Structure of the methyltransferase domain (MT-A70) of METTL3 (PDB ID: 5l6d)

There are more than 170 modifications in RNA, among which N^6^-methyladenosine (m^6^A) is the most prevalent internal modification in messenger RNA (mRNA) [1–4]. Over 7000 human transcripts harbor at least one m^6^A site, which is found within the consensus motif RRACH (where *R* = A/G, *H* = A/C/U), and most of the m^6^A sites are enriched in the coding sequence (CDS) and the 3′ untranslated region (3′UTR) of mRNA, especially around the stop codons [5, 6]. Although m^6^A was discovered more than 40 years ago [7], it failed to spark enthusiasm in this field until the identification of FTO as an m^6^A demethylase in 2011 [8], which reveals that m^6^A can be dynamically regulated and might play vital roles in development and diseases. Since then, FTO and ALKBH5, both belonging to the AlkB family of Fe(II)/a-ketoglutarate(a-KG)-dependent dioxygenases [9], were classified as m^6^A “eraser” proteins that remove m^6^A modifications from RNA (Fig. 1a and Table 1). In contrast to being removed by “eraser” proteins, m^6^A can be recognized by a set of RNA-binding proteins called m^6^A “reader” proteins that can specifically recognize and bind to m^6^A-modified transcripts. The list of m^6^A “reader” proteins is increasing (Table 1), including the YTH domain family proteins (YTHDC1/2, YTHDF1/2/3) [28, 36, 37, 41–43], the insulin-like growth factor 2 mRNA-binding proteins (IGF2BP1/2/3) [38], and the heterogeneous nuclear ribonucleoproteins (hnRNPC, hnRNPG), which were shown to mediate the regulation of RNA stability, translation efficiency, RNA splicing, and RNA exporting [44–50].

**Table 1:** m^6^A modification-related factors

Categories	Factors	Function	Ref.
Writer	METTL3/METTL14/WTAP/VIRMA/ZC3H13/RBM15	The m^6^A methyltransferase complex for the majority of m^6^A deposition	[10–14]
	METTL16	Responsible for m^6^A modification of U6 snRNA, lncRNAs, and introns of pre-mRNAs	[15–17]
	METTL5	Responsible for m^6^A modification of 18S rRNA	[18, 19]
	ZCCHC4	Responsible for m^6^A modification of 28S rRNA	[20–22]
Eraser	FTO	Demethylates m^6^A, also has activity towards m^6^A_m_ and m^1^A	[23–25]
	ALKBH5	Mainly demethylates m^6^A	[9, 26, 27]
Reader	YTHDC1	Alternative splicing and RNA export	[28, 29]
	YTHDC2	mRNA degradation and translation initiation	[30, 31]
	YTHDF1	Promotes translation	[32, 33]
	YTHDF2	Promotes RNA decay	[34, 35]
	YTHDF3	Promotes mRNAs translation and degradation	[36, 37]
	IGF2BP1/2/3	Promotes mRNA stability and translation	[38]
	hnRNPC/hnRNPG	Regulates mRNA structure and alternative splicing	[39, 40]

The enzymes catalyzing the formation of m^6^A are referred to as m^6^A “writer” proteins, first being purified as a protein complex in 1994 by Bokar et al. [51], and were further characterized in recent years as a multicomponent m^6^A methyltransferase complex (MTC) comprised of a METTL3-METTL14 heterodimer core and other binding partners (Fig. 1a), such as WTAP, ZC3H13, VIRMA, and RBM15/15B [10–14]. In addition to the MTC, other m^6^A writers have also been identified in recent years, including METTL16, METTL5, and ZCCHC4 (Fig. 1a and Table 1), which are responsible for the deposition of m^6^A into structured RNAs, such as U6 snRNA, 28S rRNA, and 18S rRNA, and in some cases, the introns of mRNA [15–22]. The MTC core component METTL3-METTL14 heterodimer catalyzes most of m^6^A methylations in mRNA, with METTL3 being the only catalytic subunit that uses S-adenosylmethionine (SAM) as the methyl donor [52–54].

The full-length METTL3 has 580 amino acids and is comprised of a zinc finger domain (ZFD) and a methyltransferase domain (Fig. 1b), both of which are needed for the enzymatic activity. Huang et al. characterized the ZFD solution structure using nuclear magnetic resonance (NMR), showing that the domain contains two tandem CCCH-type zinc fingers (ZnF1 and ZnF2) connected by an anti-parallel β-sheet (Fig. 1c), which is responsible for target recognition, specifically for binding to single-stranded RNAs containing 5′-GGACU-3′ consensus sequence [55]. The structure of the methyltransferase domain of METTL3, named MT-A70, has been determined using X-ray crystallography in a complex with the corresponding domain of METTL14 by three independent groups [52–54]. It is demonstrated that METTL14 only plays a structural role for RNA-binding and stabilization of the complex, while METTL3 is the catalytically active subunit, with a co-factor binding pocket for SAM or S-adenosylhomocysteine (SAH) [52, 53]. The crystal structure reveals that the MT-A70 domain of METTL3 is formed by a Rossman fold comprising a central, curved, eight-stranded β-sheet flanked by four α- helixes, as well as an interface loop to interact with METTL14 and two gate loops that have important roles in adenosine recognition (Fig. 1d). The conserved DPPW motif (residues 395–399) of the enzyme is located in the gate loop 1, which undergoes a significant conformational change together with gate loop 2 upon SAM/SAH binding, resulting in the closure of the co-factor binding pocket [52].

## Regulation of METTL3 expression and m^6^A deposition

**Fig. 2:**
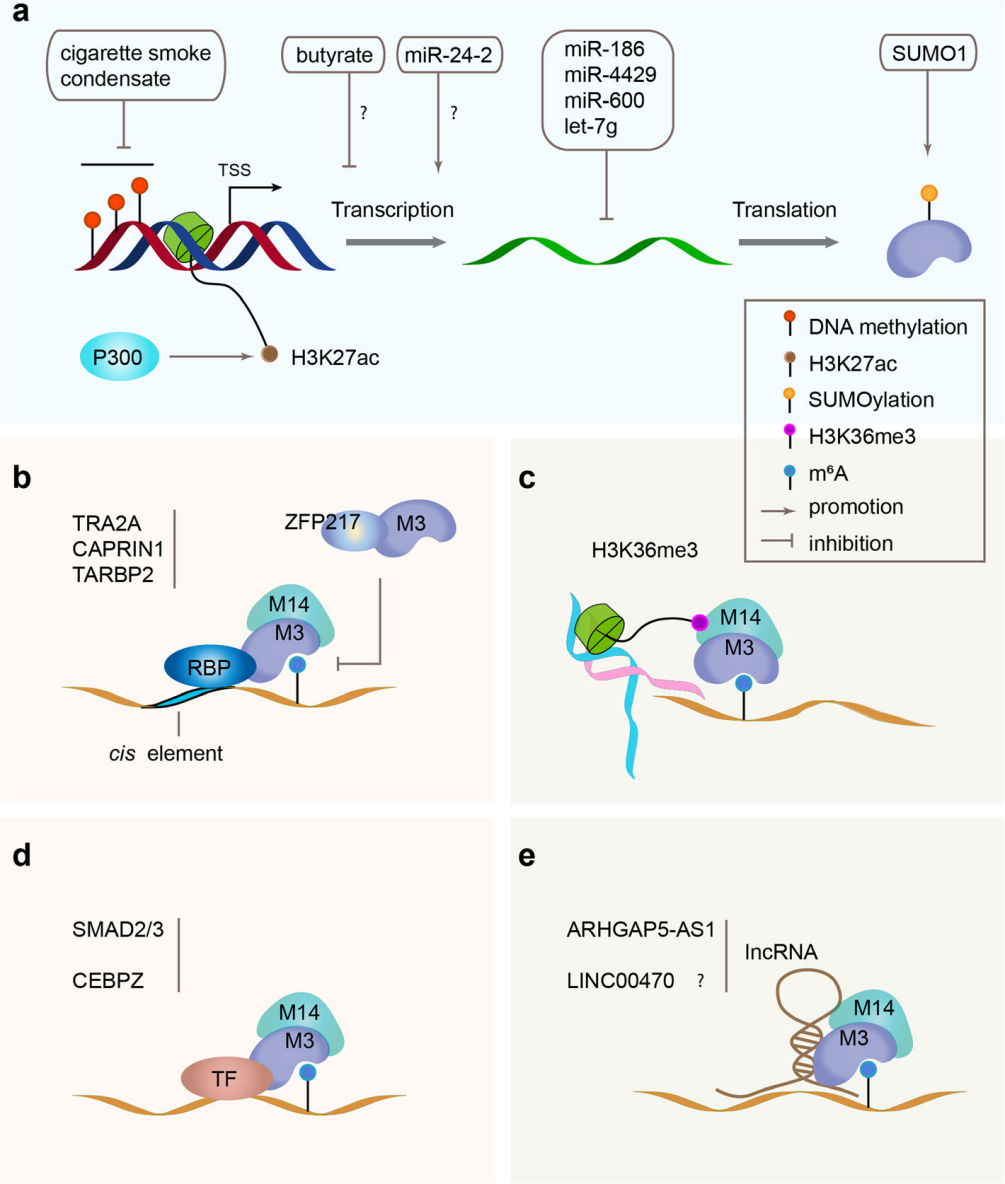
Regulation on the expression of METTL3 and its function on m^6^A deposition. **a** Multi-level regulation of METTL3. **b** Sequester or recruitment of METTL3 by RNA binding proteins (RBP). **c** Histone H3K36me3 directed deposition of m^6^A via recruiting of METTL14 and METTL3. **d** Recruitment of METTL3 by transcriptional factors (TF). **e** METTL3 guided by long non-coding RNAs (lncRNA). M3, METTL3; M14, METTL14

The expression of METTL3 is dysregulated in cancer via different mechanisms (Fig. 2a). It was demonstrated in pancreatic cancer that cigarette smoke condensate induces hypomethylation of *METTL3* promoter and subsequently the recruitment of transcription factor NFIC to induce METTL3 overexpression [56]. An intestinal microbial metabolite, butyrate, was suggested to downregulate the expression of METTL3 and inhibit the development of colorectal cancer; however, the detailed mechanism is unclear [57]. Wang et al. showed in gastric cancer that P300 mediates histone H3 acetylation at lysine 27 (H3K27ac) and promotes *METTL3* transcription [58]. It was also reported that microRNA miR-24-2 might promote *METTL3* transcription; however, the detailed mechanism remains elusive [59]. Several other microRNAs, including miR-186, miR-4429, miR-600, and let-7g [60–63], were proposed to suppress METTL3 by targeting *METTL3* mRNA. In addition, SUMOylation of METTL3 protein was reported to repress its methyltransferase activity without affecting the protein stability or localization, although the mechanism remains unclear [64].

The methyltransferase activity of METTL3 is guided in diverse manners (Fig. 2b–e). It is suggested that m^6^A deposition could be governed *in cis* via the sequence code and structure at the modified site [65]; however, the mechanism underlying was unknown. Recently, it was shown that histone H3 trimethylation at lysine 36 (H3K36me3) could guide m^6^A RNA modification co-transcriptionally through direct interaction with METTL14 and the subsequent recruitment of the MTC complex, allowing for the selective deposition of m^6^A in CDS and 3′UTR where H3K36me3 is often found [66]. Increasing efforts have also been paid to *trans* regulators; among which, zinc finger protein 217 (ZFP217) was reported to sequester METTL3 and counteracts m^6^A deposition on stemness associated transcripts [67], while the TGFβ signaling factor SMAD2/3 could recruit METTL3/14 complex to a subset of transcripts involved in early cell fate decisions [68]. Another transcriptional factor, CAATT-box-binding protein CEBPZ, was demonstrated to directly recruit METTL3 to chromatin [68]. A large-scale computational screening aimed at identifying RNA-binding proteins as cell-specific *trans* regulators of m^6^A, and validated experimentally that TRA2A and CAPRIN1 could interact with METTL3 [69]. Fish et al. found that the RNA-binding protein TARBP2 recruits METTL3 and deposits m^6^A on the introns of the target mRNA, regulating RNA splicing and stability [70]. Two long non-coding RNAs (*ARHGAP5-AS1* and LINC00470) have also been revealed to guide METTL3 to specific targets [71, 72], among which, the natural anti-sense transcript *ARHGAP5-AS1* mediates METTL3 to deposit m^6^A marks on *ARHGAP5* mRNA, promoting the mRNA stabilization and inducing chemoresistance [71].

## METTL3 functions as an m^6^A methyltransferase in cancer

Accumulating evidence in recent years has demonstrated that METTL3 plays critical roles in cancer as an m^6^A methyltransferase, either as an oncogene or a tumor suppressor, as summarized in Table 2.

**Table 2:** Roles of METTL3 as an m^6^A methyltransferase in human cancers

Role	Cancer type	Regulator	Targets	Molecular mechanism	Cellular function	Ref.
Oncogene	Acute myeloid leukemia		*MYC*, *BCL2*, *PTEN*	Promote translation	Differentiation, apoptosis	[73]
		CEBPZ	*SP1*, *SP2*	Promote translation	Cell cycle regulation, differentiation	[74]
	Breast cancer	let-7g	*HBXIP*	Promote translation (?)	Cell proliferation	[63]
			*BCL2*	Promote translation	Proliferation, apoptosis	[75]
	Liver cancer		*SOCS2*	RNA decay by YTHDF2	Proliferation, migration	[76]
			*RDM1*		Proliferation	[77]
			*LINC00958*	RNA stabilization	Lipogenesis, proliferation, migration, invasion	[78]
		SUMO1	*Snail*	RNA stabilization	Metastasis	[79]
			*Snail*	promote translation by YTHDF1	EMT	[80]
		miR24-2	*miR-6097*, *Pim1*		Tumor growth	[59]
			*CTNNB1*	RNA stabilization	Tumor growth	[81]
		miR-186	*Wnt/β-catenin*		Proliferation, migration, invasion	[60]
	Glioblastoma		*SOX2*	RNA stabilization by ELAVL1	Dedifferentiation	[82]
			*SRSFs*	Nonsense-mediated mRNA decay (NMD) by YTHDC1	Tumor growth and progression	[83]
	Bladder cancer		*miR-221/222*	miRNA maturation	Proliferation	[84]
			*AFF4*, *RELA*, *IKBKB*, *MYC*		Proliferation, apoptosis	[85]
			*ITGA6*	Promote translation by YTHDF1/3	Adhesion, migration, invasion	[86]
		chemical carcinogenesis	*CPCP*	Promote translation	Malignant transformation	[87]
			*SETD7*, *KLF4*	RNA decay by YTHDF2	Proliferation, metastasis	[88]
	Gastric cancer	miR-4429	*SEC62*	RNA stabilization by IGF2BP1	Proliferation, apoptosis	[62]
			AKT signaling pathway		Proliferation, migration, invasion	[89]
		CBP/P300-mediated H3K27ac	*HDGF*	RNA stabilization by IGF2BP3	Tumor angiogenesis and glycolysis	[58]
			*ZMYM1*	RNA stabilization by ELAVL1	EMT	[90]
		LncRNA ARHGAP5-AS1	*ARHGAP5 mRNA*	RNA stabilization	Chemoresistance	[71]
			MYC target genes		Proliferation, migration, invasion	[91]
		LINC00470	*PTEN*	RNA decay by YTHDF2	Proliferation, migration, invasion	[72]
	Prostate cancer		*GLI1*		Proliferation, migration, apoptosis,	[92]
			*MYC*		Proliferation, migration, invasion.	[93]
			*ITGB1*	RNA stabilization	Cell adhesion	[94]
	Lung cancer	miR-600	*β-catenin*, *TAZ*, *EGFR*, *DNMT3A*		Proliferation, metastasis, apoptosis	[61]
			*miR-25-3p*	miRNA maturation	Brain metastasis	[95]
		TGF-β	*JUNB*	RNA stabilization	EMT	[96]
			*YAP*, *MALAT1*	promote translation by YTHDF1/3; RNA stabilization by YTHDF3	Drug resistance and metastasis	[97]
	Colorectal cancer		*SOX2*	RNA stabilization by IGF2BP2	Tumorigenesis, metastasis	[98]
			*lncRNA RP11*	Nucleus accumulation	Metastasis	[99]
			*miR-1246*	miRNA maturation	Metastasis	[100]
			*HK2, SLC2A1*	RNA stabilization by IGF2BP2/3	Activation of the glycolysis pathway	[101]
		Butyrate	*CCNE1*	RNA stabilization	Proliferation	[57]
			*circNSUN2*	Exporting to cytoplasm	Metastasis	[102]
			*CBX8*	RNA stabilization by IGF2BP1	Stemness	[103]
	Pancreatic cancer	NFIC	*miR-25-3p*	miRNA maturation	Proliferation, metastasis	[56]
					Proliferation, invasion	[104]
	Osteosarcoma		*LEF1*		Proliferation, migration, invasion	[105]
			*DRG1*	RNA stabilization by ELAVL1	Proliferation	[106]
			*ATAD2*		Proliferation, migration, invasion	[107]
	Oral squamous cell carcinoma		*MYC*	RNA stabilization by YTHDF1	Proliferation, invasion, migration	[108]
	Thyroid carcinoma		*TCF1*	RNA stabilization by IGF2BP2	Activating the Wnt pathway, migration	[109]
	Uveal melanoma		*c-Met*		Proliferation, migration, invasion	[110]
	Ovarian cancer		*AKT*		Proliferation	[111]
	Head and neck squamous cell carcinoma		*LNCAROD*	RNA stabilization	Proliferation, mobility	[112]
	Cutaneous squamous cell carcinoma		*ΔNp63*		Proliferation	[113]
	Nasopharyngeal carcinoma		*ZNF750*		Apoptosis	[114]
Tumor suppressor	Renal cell carcinoma				Proliferation, migration, apoptosis,	[115]
	Glioblastoma		*ADAM19*		Self-renewal	[116]
	Endometrial cancer		*PHLPP2*, *mTORC2*	Promote translation by YTHDF1; RNA decay by YTHDF2	Proliferation	[117]
	Ocular melanoma		*HINT2*	Promote translation by YTHDF1	Proliferation, apoptosis	[118]
	Colorectal cancer		p38/ERK pathways		Proliferation, migration, invasion	[119]
	Bladder cancer					[120]

### METTL3 as an oncogene

**Fig. 3:**
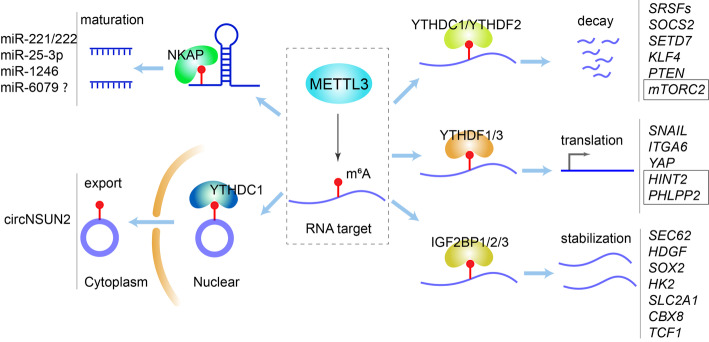
Molecular mechanisms underlying regulation of METTL3 on target genes in cancers. METTL3 methylates target transcripts, and the resulting m^6^A marks recruit m^6^A reader proteins to determine RNA fate. The target genes of METTL3 are in italics, with those involved in the tumor-suppressive function of METTL3 being boxed. CRC, colorectal cancer; BCa, bladder cancer, NSCLC, non-small cell lung cancer; PDAC, pancreatic ductal adenocarcinoma; HCC, hepatocellular carcinoma; GBM, glioblastoma; GC, gastric cancer; AML, acute myeloid leukemia; OM, ocular melanoma; EC, endometrial cancer; TC, thyroid carcinoma

In most cases, METTL3 was reported as an oncogene to promote the initiation and development of a variety of cancers, including hematopoietic malignancies and solid tumors, through depositing m^6^A modification on critical transcripts (Fig. 3).

### Acute myeloid leukemia

Acute myeloid leukemia (AML) is one of the most common hematopoietic malignancies with diverse genetic and molecular abnormalities, in which the hematopoietic stem and progenitor cells (HSPCs) retain the self-renewal capacity, while the myeloid differentiation capacity is hampered [121–123]. Vu et al. found that METTL3 was more abundant in AML cells than in normal HSPCs, and when overexpressed, the wild-type but not the catalytically inactive form of METTL3 could inhibit the differentiation of HSPCs. In AML cells, depletion of METTL3 induced cell differentiation and apoptosis and delayed leukemia progression. The authors further demonstrated that METTL3 mediated m^6^A modification on *MYC*, *BCL2*, and *PTEN* mRNAs and promoted their translation [73].

By performing two independent CRISPR screens, Barbieri et al. showed that METTL3 was necessary for AML cell survival and further revealed that the CAATT-box-binding protein CEBPZ was required for recruitment of METTL3 to chromatin. Promoter bound METTL3 introduces m^6^A modification within the coding region of *SP1* and *SP2* transcripts and enhances their translation, thus promoting cell proliferation and AML maintenance [74].

### Liver cancer

Liver cancer is the fourth most common cause of cancer-related death worldwide, among which, hepatocellular carcinoma (HCC) accounts for the majority of cases [124], and the genetic and epigenetic alterations including m^6^A deregulation have been widely investigated [125, 126].

It was reported that METTL3 was significantly upregulated in HCC and was associated with shorter overall survival in HCC patients, while METTL3 depletion significantly inhibited HCC tumorigenicity and metastasis. Mechanistically, METTL3 mediates m^6^A modification of *suppressor of cytokine signaling 2* (*SOCS2*) and promotes its mRNA degradation via a YTHDF2-dependent pathway [76]. In accordance with this, however, via an alternative mechanism, Lin et al. showed that METTL3 regulated the epithelial-mesenchymal transition (EMT) of HCC cells by methylating the CDS of *Snail* and triggering polysome-mediated translation [80]. A recent study showed that m^6^A modification by METTL3 on *RAD52 motif 1* (*RDM1*) mRNA represses its expression and increases HCC cell proliferation via p53 and Ras/Raf/ERK pathways [77]. METTL3 can also promote HCC by methylating non-coding RNAs. Zuo et al. revealed that METTL3-mediated m^6^A modification promoted the stabilization of LINC00958, which sponged miR-3619-5p to upregulate *hepatoma-derived growth factor* (*HDGF*) and thereby facilitating HCC lipogenesis and progression [78].

As the most common pediatric liver cancer, hepatoblastoma (HB) has also been reported to have aberrant m^6^A modification [60, 81]. Liu et al. found that METTL3 was significantly upregulated in HB, inducing enhanced m^6^A modification across the whole transcriptome. They further identified *CTNNB1*, a key component of the Wnt/β-catenin signaling pathway, as a target of METTL3 [81]. Cui et al. proposed METTL3 as an independent prognostic factor in HB patients. They showed that knockdown of METTL3 dramatically suppressed HB cell proliferation, migration, and invasion via regulating Wnt/β-catenin pathway-associated proteins [60].

### Gastric cancer

Gastric cancer (GC) is the fifth most common cancer and the third most lethal malignancy worldwide [127]. The study from Wang et al. showed that the m^6^A level significantly increased in GC cells owing to P300-mediated METTL3 overexpression. Specifically, elevated m^6^A level of *HDGF* mRNA promoted its stability via the binding of IGF2BP3, and the upregulated HDGF activated *GLUT4* and *ENO2* expression to promote glycolysis and eventually tumor growth and metastasis of GC cells [58]. Consistently, another two studies also reported that the upregulation of METTL3 in GC promoted cell proliferation and migration via affecting AKT signaling pathway and MYC target genes, respectively [89, 91].

The elevated METTL3 level was correlated with tumor survival and recurrence of GC patients. *Zinc finger MYM-type containing 1* (*ZMYM1*) was identified as a target of METTL3 and the stability of m^6^A-modified *ZMYM1* was enhanced by HuR (also known as ELAVL1) binding, leading to E-cadherin repression and EMT progression [90]. *SEC62* is another target of METTL3 that is stabilized by IGF2BP1 in an m^6^A-dependent manner. The upregulation of *SEC62* in GC promotes cell proliferation [62].

### Lung cancer

Lung cancer is the leading cause of cancer incidence and mortality worldwide [127], and non-small cell lung cancer (NSCLC) comprises the most majority (~ 85%) of all lung cancers. Jin et al. demonstrated that METTL3 increases m^6^A modification of both *YAP* and lncRNA *MALAT1*, which induces drug resistance and metastasis of NSCLC cells through diverse pathways [97]. During TGFβ-induced EMT of NSCLC cells, both *METTL3* expression and total m^6^A level were upregulated [96]. *METTL3* knockdown decreased the m^6^A modification of *JUNB*, one of the important EMT regulators, leading to mRNA destabilization [96]. The precursor miR-143-3p was reported as a direct target of METTL3; increased m^6^A level of the precursor miRNA promotes its maturation, which induces invasion of NSCLC by targeting 3′UTR of *vasohibin-1 (VASH1*) to inhibit its expression [95].

### Colorectal cancer

Colorectal cancer (CRC) is a complex and heterogeneous carcinoma tightly related to dietary and lifestyle factors, and a number of studies have uncovered genetic alterations and epigenetic dysregulation involved in CRC [128]. It is reported in recent years that m^6^A RNA modification also plays key roles in CRC progression. Li et al. found that the expression of METTL3 was higher in CRC metastatic tissues and was associated with a poor prognosis. They demonstrated that *SRY-box 2* (*SOX2*) was a target of METTL3, and the m^6^A in the coding region of *SOX2* mRNA was recognized by IGF2BP2 to protect the mRNA from decay [98]. Another study showed that METTL3 methylated *CBX8* mRNA to enhance its stability in an IGF2BP1-dependent manner and that the upregulated CBX8 could promote stemness and suppress chemosensitivity of CRC cells through regulating *LGR5* expression [103]. A role of METTL3 in regulating cell metabolism in CRC was also reported, where METTL3 regulates the glycolysis pathway through depositing m^6^A modification on the UTR of *HK2* and *SLC2A1* (*GLUT1*) [101].

Three studies highlight the role of non-coding RNAs in mediating the function of METTL3 in CRC. Wu et al. reported that m^6^A methylation of lncRNA *RP11* by METTL3 increased its nuclear accumulation, which triggers the migration and invasion of CRC cells via post-translational upregulation of Zeb1 [99]. Peng et al. found that overexpressed METTL3 in CRC could methylate pri-miR-1246 and promote miRNA maturation to downregulate SPRED2, leading to tumor metastasis [100]. m^6^A modification of the circular RNA *circNSUN2* increases its export to the cytoplasm, where it stabilizes *HMGA2* mRNA to promote CRC metastasis [102].

### Bladder cancer

Bladder cancer (BCa) is more common in men, with a 4 times higher incidence and mortality rate than in women globally [127]. Two groups found that METTL3 was upregulated in BCa patient samples and further demonstrated that METTL3-mediated m^6^A modification in target transcripts, such as *CDCP1* and *ITGA6*, promoted mRNA translation via YTHDF1/3 binding [86, 87]. Other mRNA transcripts, including *AFF4*, *IKBKB*, *RELA*, and *MYC*, were revealed to be targets of METTL3 and mediated the role of METTL3 in promoting cell proliferation, invasion, and survival [85], whereas *SETD7* and *KLF4* tumor suppressors were negatively regulated by METTL3 in a YTHDF2-dependent manner [88]. Recently, a study by Han et al. showed that METTL3-mediated m^6^A modification in pri-miR221/222 facilitates miRNA maturation, resulting in the reduction of *PTEN*, which ultimately leads to BCa cell proliferation [84].

### Pancreatic cancer

Pancreatic cancer, primarily pancreatic ductal adenocarcinoma (PDAC), is a highly lethal disease with mortality closely parallel with incidence [129]. It was suggested that a high level of METTL3 expression was associated with a high pathological stage in PDAC [104], as well as chemo- and radio-resistance in pancreatic cancer cells [130]. Zhang et al. conducted an elaborate study demonstrating that cigarette smoke condensate induced METTL3 expression and increased m^6^A modification on the oncogenic primary miR-25, leading to the enhanced maturation of the miRNA and the activation of AKT signaling in pancreatic duct epithelial cells to provoke malignant transformation [56].

### Glioblastoma

Glioblastoma is an aggressive malignancy of astrocytes with high molecular heterogeneity and poor prognosis [131]. Visvanathan et al. reported that METTL3-mediated m^6^A modification on *SOX2* mRNA plays crucial roles in glioma stem-like cells (GSCs) maintenance and dedifferentiation [82]. In consistence with this, Li et al. found that elevated expression of METTL3 was highly correlated with the clinical aggressiveness of malignant gliomas. They further showed that knockdown of METTL3 decreases m^6^A modification of the splicing factor *SRSF*, leading to YTHDC1-dependent nonsense-mediated mRNA decay of the *SRSF* transcripts and alternative splicing isoform switches in glioblastoma [83].

### Prostate cancer

Prostate cancer (PCa) ranks as the second most frequent cancer worldwide in men, and it is the most lethal malignancy among men in 46 countries; however, relatively little is known regarding its etiology [127]. Cai et al. found that *METTL3* is overexpressed in PCa cells and that *METTL3* silencing decreases m^6^A modification and downregulates *GLI1*, an important component of the hedgehog pathway, which induces cell apoptosis [92]. Yuan et al. proposed *METTL3* upregulation as a poor prognostic factor in PCa patients and revealed *MYC* as a METTL3 target [93]. In a recent report, Li et al. showed that *METTL3* is upregulated in PCa tissues, especially those with bone metastasis. Their data suggested that METLT3-mediated m^6^A in *Integrin β1* (*ITGB1*) stabilizes the mRNA via specific binding of HuR, which enhances cell motility and bone metastasis [94].

### Breast cancer

A positive feedback mechanism was suggested in the progression of breast cancer, where upregulated METTL3 promoted the expression of *hepatitis B X-interacting protein* (*HBXIP*) in an m^6^A-dependent manner, and HBXIP inhibited miRNA let-7 g, a *METTL3* negative regulator, thereby maintaining the high expression level of METTL3 and the accelerated cell proliferation in breast cancer [63]. Wang et al. also reported *METTL3* overexpression in breast cancer and identified *BCL2* as a target of METTL3, demonstrating that *Bcl-2* translation was promoted owing to elevated m^6^A modification in the mRNA, which eventually promoted cancer cell proliferation [75].

### Other cancers

In addition to the aforementioned cancer types, METTL3 also plays an oncogenic role in other types of cancer (Table 2). LEF1, ATAD2, c-Met, AKT, ΔNp63, and ZNF750 have been suggested as oncogenes in association with METTL3 with uncharacterized pathways [105, 107, 110, 111, 113, 114]. Several mRNAs, including *DRG1*, *MYC*, and *TCF1*, as well as non-coding RNA *lncAROD*, have been suggested as METTL3 targets that are stabilized in a m^6^A-dependent manner, resulting in cell proliferation and/or migration in osteosarcoma, oral squamous cell carcinoma (OSCC), thyroid carcinoma, and head and neck squamous cell carcinoma (HNSCC), respectively [106, 108, 109, 112]. It is noteworthy that in the case of OSCC, the authors concluded that the m^6^A modification in *MYC* enhanced the mRNA stability mediated by YTHDF1 [108], which was a different mechanism from that in other reports and highlighted the functional complexity of the protein.

### METTL3 as a tumor suppressor

While METTL3 exhibits oncogenic functions in most cancer types, it was also reported as a tumor suppressor in some cases [132]. Li et al. detected lower expression of METTL3 in renal cell carcinoma (RCC) tissues compared with adjacent non-tumor tissues and also showed that higher expression of METTL3 might predict better survival outcome of RCC patients, possibly by promoting cell cycle arrest in G1 phase and thus suppressing tumor growth [115]. Based on a novel statistical model and the following experimental validation, Zhao et al. also identified METTL3 as a tumor suppressor gene in bladder cancer and showed that somatic mutations in METTL3 may promote cancer cell growth [120]. Cui et al. showed that the self-renewal of glioblastoma stem cell (GSC) was regulated by m^6^A mRNA modification and that knocking-down of METTL3 significantly promoted tumor progression and shortened the lifespan of GSC-grafted animals [116]. A similar conclusion was made by Deng et al. in CRC, where they found that METTL3 suppresses cell proliferation, migration, and invasion through p38/ERK pathways and thus is associated with longer survival time [119].

Hypomethylation of m^6^A resulting from decreased METTL3 expression was also observed in some cancers. A study from Jia and colleagues reported that decreased m^6^A level in ocular melanoma due to downregulation of METTL3 and upregulation of ALKBH5 predicted earlier recurrence and enhanced aggressiveness and also showed that METTL3-mediated m^6^A modification promoted the translation of *HINT2*, a tumor suppressor gene, in a YTHDF1-dependent mechanism [118]. Liu et al. depicted a more complicate scenario, in which they found that about 70% of endometrial tumors exhibit m^6^A hypomethylation, probably due to a hotspot mutation in *METTL14* or reduced expression of *METTL3*, which resulted in downregulation of the negative AKT regulator PHLPP2 and upregulation of the positive AKT regulator mTORC2 [117].

## METTL3 promotes cancer independent of catalytic activity

Elevated expression of METTL3 was detected in lung cancer cells, which was associated with cancer cell growth, survival, and invasion [133]. In-depth investigation found that METTL3 promoted the translation of certain oncogenic transcripts such as EGFR and TAZ, independent of its catalytic activity, but through recruiting eukaryotic translation initiation factor 3 (eIF3) to the translation initiation complex [133]. In a following report from the same group, the authors further revealed that the N-terminal fragment of METTL3 (1–200 amino acids) interacted directly with the eIF3 subunit h (eIF3h) and that the mutated METTL3 (A155P) abrogated the interaction and the ability to promote mRNA translation was severely compromised [134]. The authors thus proposed a novel “mRNA looping” mechanism, in which METTL3 binds to the m^6^A-modified 3′UTR of target mRNA and then recruits eIF3h as well as translation initiation factors such as CBP800 and eIF4e, thus facilitating the ribosome recycling and promoting translation efficiency [133, 134].

A recent work by Hua et al. also presented a similar mechanism, in which METTL3 promoted translation of the receptor tyrosine kinase AXL, independent of its methyltransferase activity. The authors also showed that upregulated METTL3 in ovarian carcinoma was significantly associated with tumor grade and overall survival rate [135]. It is noteworthy that METTL3 only binds to approximately 22% of the m^6^A sites [14], suggesting a mechanism of selectivity of METTL3 towards its targets for translational regulation, which needs to be further elucidated in the future.

## Targeting of METTL3 for potential clinical application

Based on the emerging data on the roles and the molecular mechanisms in cancer, m^6^A regulators have attracted growing investigation as therapeutic targets [136]. As discussed above, METTL3 plays both oncogenic and tumor-suppressive roles in human cancers. Although still limited, activators and inhibitors targeting METTL3 have been reported recently. A structure-based virtual screening of compound databases carried out by Selberg et al. discovered four small molecules displaying the potency of enhancing the activity of the METTL3-METTL14 complex, among which the most effective one increased the relative m^6^A level by 21.4 ± 12.9% in the following cellular assays [137]. Simultaneous docking analysis showed that the compound interacted with SAM in close proximity in the active center of METTL3, which might increase the binding affinity of SAM and also lower the energy barrier of the substrate RNA methylation reaction [137].

**Fig. 4:**
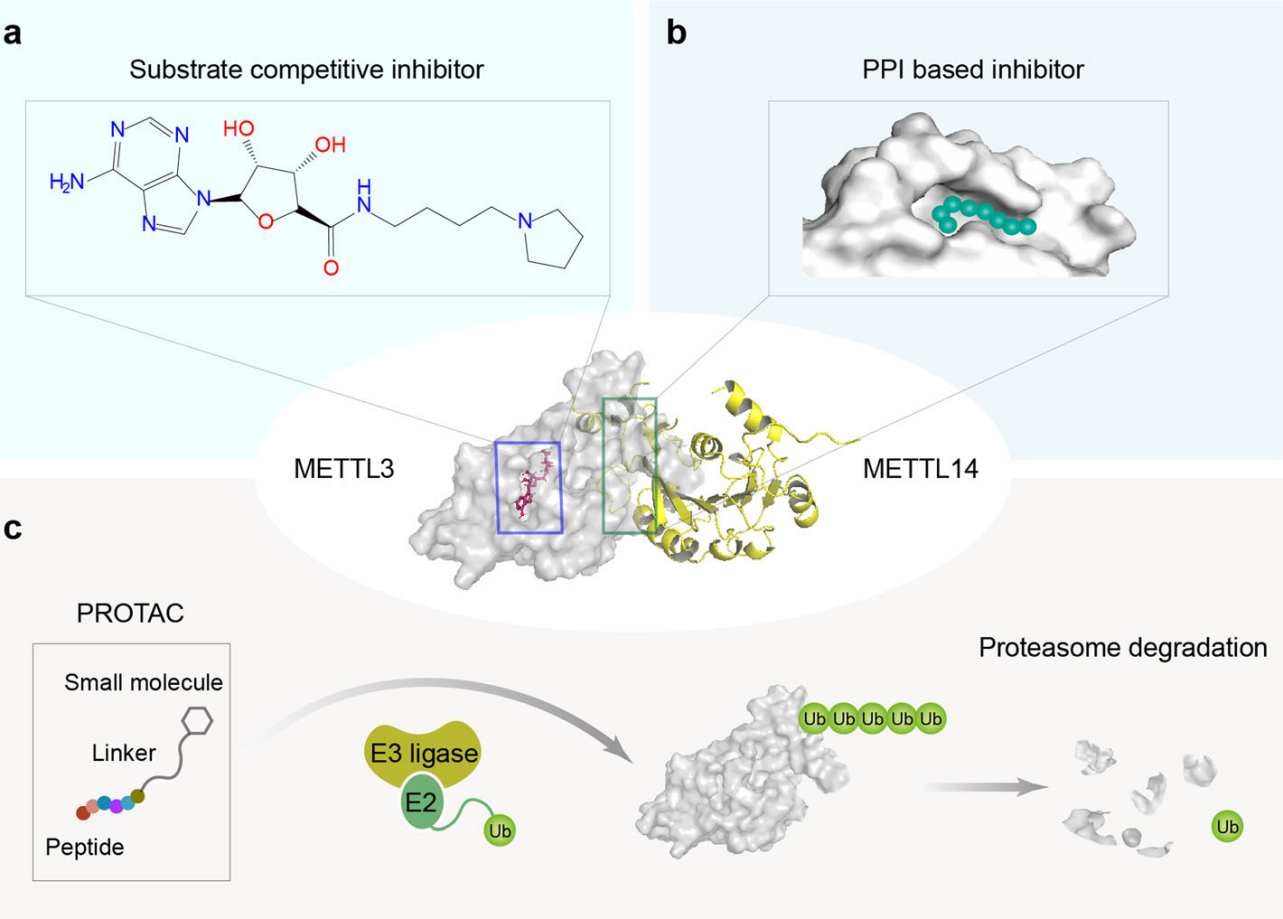
Potential strategies for targeting of METTL3 in cancer. **a** A reported substrate competitive inhibitor of METTL3. **b** Protein-protein interaction (PPI)-based drug design. **c** Proteolysis-targeting chimera (PROTAC) for the degradation of METTL3 protein

Another study performed by Bedi et al. used co-factor mimicking approach to screen a library of 4000 adenosine analogs and derivatives and identified seven compounds showing diverse activities in two in vitro assays; among them, one compound (illustrated in Fig. 4a) has the most favorable inhibitory potency (IC_50_ = 8.7 μM) [138]. This is the first study reporting METTL3 inhibitors; however, several issues remain to be addressed. First, there is a lack of cellular activity data; thus, the actual effect of the compounds on m^6^A level is unknown. Second, the potential use of the compounds needs to be carefully evaluated because of the poor cell permeability and pharmacokinetics of adenosine analogs. Third, the co-factor binding pockets are common to the wide range of Rossmann fold enzymes; therefore, the selectivity of the inhibitors also requires further investigation.

As discussed above, the oncogenic function of METTL3 relies on the heterodimer structure formed with METTL14 in most cases; therefore, it will be a reasonable option to design inhibitors based on protein-protein interaction (PPI) strategy (Fig. 4b), as having been successfully applied in other drug targets [139]. Proteolysis targeting chimera (PROTAC) is another emerging biotechnology to degrade a wide range of proteins specifically [140], which is a bifunctional molecule consisting of a ligand of the target protein and a covalently linked ligand of an E3 ubiquitin ligase (Fig. 4c). Whether this strategy could be applied for degrading METTL3 in cancers warrants further investigation. In addition, structural analysis suggested that the substrate-binding site and SAM-binding site of METTL3 are merged into a large pocket [141]; therefore, the development of bisubstrate inhibitors that simultaneously occupy both of the binding sites might be an alternative strategy for future METTL3 targeting.

Overall, targeting of METTL3 for clinical application is still in its infancy. With the increasing knowledge regarding the regulation, functions, and mechanisms of METTL3 in cancer, it is promising to develop METTL3 targeted agents in the near future.

## Conclusions and perspectives

It is widely accepted that several biological capabilities are regarded as hallmarks of cancer, including uncontrolled proliferation, cell death resistance, angiogenesis, invasion and metastasis, metabolism dysregulation, and immune escape [142]. The accumulating studies in recent years have revealed diverse pathways in cancers that are affected by METTL3, mostly focused on cell proliferation, cell death resistance, invasion, and metastasis (Table 2). However, emerging evidence also suggests that METTL3 plays vital roles in other biological processes, such as angiogenesis [58] and metabolism dysregulation [58, 101]. A recent publication showed that m^6^A modification of viral RNA enables the virus to avoid detection by innate immunity [143]; thus, there is a possibility that METTL3 might also mediate immune escape in cancers. Further studies are warranted to fully uncover the roles and mechanisms of METTL3 in affecting every hallmark of cancer.

It should be emphasized that although this review focuses mostly on METTL3, other components of the MTC, especially METTL14, should be taken into account when studying the m^6^A-dependent function of METTL3 in cancer. It is reasonably thought that proteins in the MTC should exhibit similar roles in the same pathological conditions; however, this is not always the case as reported. For example, METTL14 plays similar roles as METTL3 in some types of cancer, such as AML [122, 144, 145], breast cancer [146], and endometrial cancer [117], but exhibits opposite functions in other cancers, such as liver cancer [147, 148]. How could components in the same protein complex play opposite roles in the same cancer type needs to be carefully investigated and addressed. It is also noticeable that in glioblastoma, bladder cancer, and colorectal cancer, METTL3 was reported to play either oncogenic or/and tumor-suppressive functions by different groups, which may be explained by tumor heterogeneity and/or different model systems used for the study, and more further comprehensive and detailed studies are warranted to gain a better view.

In conclusion, METTL3 affects a broad range of biological processes and plays diverse roles in cancers, either dependent or independent of its methyltransferase activity, while the therapeutic targeting of METTL3 is just in the early stage. Continuing efforts are still needed to design and optimize strategies for targeting of METTL3 for cancer treatment.

## Data Availability

Not applicable.

## Abbreviations

m^6^A: N6-methyladenosine
METTL3: Methyltransferase-like 3
METTL14: Methyltransferase-like 14
ALKBH5: AlkB Homolog 5
FTO: Fat mass and obesity associated
YTHDC: YT521-B homology domain containing
YTHDF: YT521-B homology domain family
IGF2BP: Insulin-like growth factor 2 mRNA-binding protein
HNRNPC: Heterogeneous nuclear ribonucleoproteins C
HNRNPG: Heterogeneous nuclear ribonucleoproteins G
MTC: m^6^A methyltransferase complex
NMR: Nuclear magnetic resonance
AML: Acute myeloid leukemia
HSPCs: Hematopoietic stem and progenitor cells
HCC: Hepatocellular carcinoma
CRC: Colorectal cancer
GC: Gastric cancer
NSCLC: Non-small cell lung cancer
BCa: Bladder cancer
PDAC: Pancreatic ductal adenocarcinoma
GSCs: Glioma stem-like cells
PCa: Prostate cancer
OSCC: Oral squamous cell carcinoma
HNSCC: Head and neck squamous cell carcinoma
RCC: Renal cell carcinoma
eIF3: Eukaryotic translation initiation factor 3
PROTAC: Proteolysis targeting chimera
